# A capillary-based multiplexed isothermal nucleic acid-based test for sexually transmitted diseases in patients†
†Electronic supplementary information (ESI) available: Additional methods and experimental sections. See DOI: 10.1039/c6cc05679b



**DOI:** 10.1039/c6cc05679b

**Published:** 2016-09-08

**Authors:** Gaolian Xu, Hang Zhao, Jonathan M. Cooper, Julien Reboud

**Affiliations:** a Division of Biomedical Engineering , School of Engineering , University of Glasgow , Oakfield Avenue , Rankine Building , G12 8LT Glasgow , UK . Email: Julien.Reboud@glasgow.ac.uk

## Abstract

We demonstrate a multiplexed loop mediated isothermal amplification (LAMP) assay for infectious disease diagnostics, where the analytical process flow of target pathogens genomic DNA is performed manually by moving magnetic beads through a series of plugs in a capillary. Heat is provided by a water bath and the results are read by the naked eye, enabling applications in low resource settings.

## Abstract 2

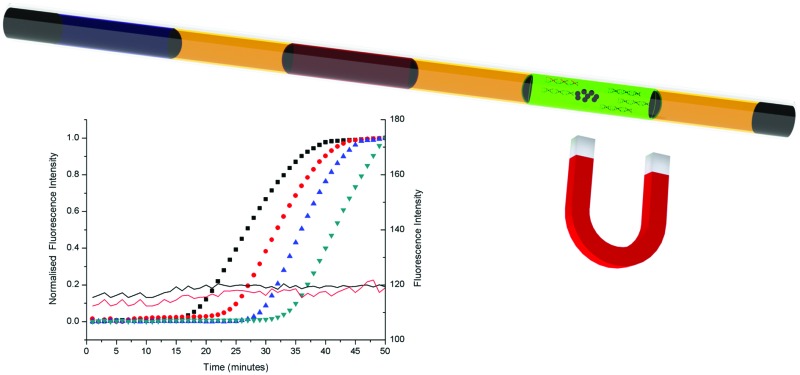
A sample-to-answer nucleic-acid based assay, processed magnetically inside capillaries, enables multiplexing analysis in low resource settings.

Recently, rapid, high performance nucleic acid testing (NAT) based diagnostic technologies have shown the ability to detect infections early.^1^ However, the existing technologies for the diagnosis of sexually transmitted diseases, such as chlamydia and gonorrhoea, suffer from limitations in speed, cost, and the significant user expertise required.^1^ The most widely used technology is based on the polymerase chain reaction (PCR), which attains high performance, but is difficult to carry out in resource limited areas.^1^ As an alternative, low cost nucleic acid based assays using isothermal amplification have the ability to provide platforms for point-of-care (POC) diagnostics in both low and high resource settings, due to their high sensitivities and specificities.^2^

Isothermal amplifications, such as the loop-mediated systems (LAMP), also offer the capability of multiplexing,^3,4^ which in turn can be used to inform treatment in *e.g.* infectious diseases (where the relative sensitivities to specific drug regimes are often determined genetically). Here we focus on detecting two sexually-transmitted pathogens, *Chlamydia trachomatis* and *Neisseria gonorrhoea*, which are prevalent in both high as well as low and middle income countries (LMICs).^5^ The LAMP reaction generally requires six DNA primers for each target to be detected, limiting the possibilities of simultaneously amplifying multiple targets in single reactions, due to the high level of potential cross-talk between the reactions. This constraint has restricted the development of multiplexing POC devices, since adding independent reactions to a single device in turn increases practical challenges linked to reagent mixing, analysis and temperature control.

Here we show that by exploiting capillary-based platforms for multiplexing and in line sample processing,^6–8^ through the use of plugs separated by an immiscible phase, we are able to demonstrate a powerless, low-cost, easy-to-use sample-to-answer multiplexed LAMP assay. Our new platform circumvents the requirements for external fluid actuation and electronic thermal control systems, potentially facilitating its deployment as a POC device in resource-limited settings.

The advantages of using capillary-based platforms, such as ease of use and multiplexing DNA amplification reactions of extracted samples, have been realised previously,^6^ while single target DNA extraction was successfully demonstrated,^7^ and DNA lysis incorporated only recently within a similar format using a FTA card (Flinders Technology Associates), but requiring multiple pipetting steps.^8^ In this paper, we now go beyond the current state of the art and show the complete nucleic-acid processing (from crude samples to the answer) in a multiplexed and easy-to-use format.

To achieve this, we integrated three different capabilities into the capillary-based system, namely: manual sample manipulation with magnetic beads to extract DNA from complex samples; tolerance to temperature variability (enabling the use of heated water to drive the reaction); and a visual detection of a calcein fluorescent probe on the amplified product, illuminated by a UV flashlight.^9^

Previously, magnetic beads have been integrated within microfluidic devices for DNA separation and purification,^10,11^ providing a simple manual method for collection of bound probes or targets without the need for centrifugation or filtration and with no requirement for external power.^12^ They also advantageously replace previously used FTA cards,^8^ which are difficult to handle and limit the applicability of previously developed tip-based systems to trained experts. In the assay configuration that we describe, the magnetic beads served as solid carriers for DNA (Fig. 1). By moving a handheld magnet, the DNA passed through a series of discrete process steps, defined by a series of plugs within the capillary, which enabled us to purify and enrich the sample, removing any inhibitors prior to the amplification reaction. The series of plugs simply comprised different aqueous phases separated by mineral oil,^13^ which also served to prevent evaporation during the amplification step at 60 °C. Multiplexing was enabled by sequentially adding separate plugs containing different reaction mixtures specific to each of the targets.

**Fig. 1:**
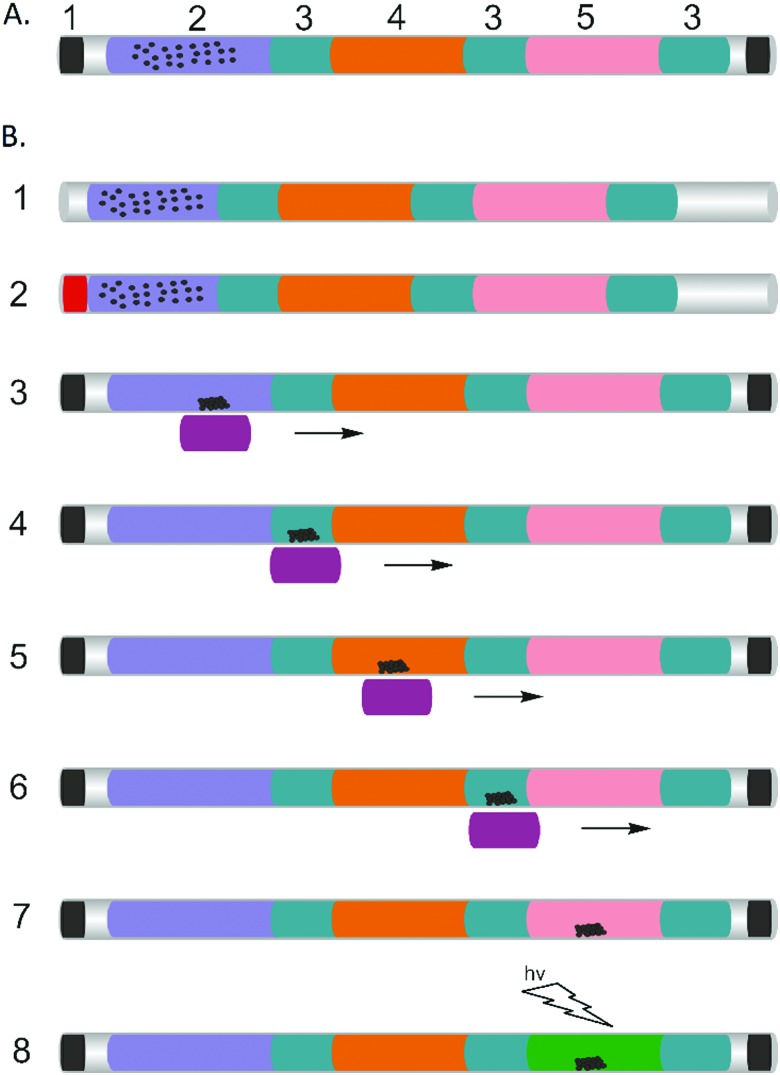
(A) The integrated capillary LAMP system with preloaded reagents after the addition of the sample. (1) Epoxy glue sealant; (2) lysis buffer and magnetic beads (black spots) to release and capture DNA; (3) mineral oil; (4) washing buffer; (5) LAMP reaction mix. (B) Process sequence: (1) pre-filled capillary (2) sample (red) injection, lysis and DNA binding; (3) sealing with epoxy glue (black) and aggregation of magnetic beads; (4–6) passing through mineral oil into the washing buffer to remove impurities; (7) elution of DNA in the LAMP reaction mix and LAMP amplification (by heating to 60–64 °C); (8) illumination using a UV flashlight to reveal the assay results.

The whole process was completed within 60 min. 1 μl of sample (in this case a model target DNA, at different concentrations in PBS) was manually pipetted into the pre-filled capillary (Fig. 1 and Experimental methods in the ESI†), where it was mixed with lysis buffer, which also contained the magnetic beads. Epoxy was used to seal the ends of the capillary to prevent spillage and the capillary was left at room temperature for 3 minutes to lyse the cells and capture the DNA on the beads (in a low pH and high ionic strength solution). The beads (carrying the DNA) were then subsequently moved through the mineral oil separator into the washing section (Fig. 1B(3–4) and 2A), where they were held for 1 min to remove residues and contaminants from the beads, while retaining the DNA.^14^ A single wash step was sufficient to purify the DNA to a level that enabled the LAMP reactions to proceed efficiently. The magnet was moved further along the capillary, through an oil separator into the LAMP reaction mixture, where the amplification reaction was performed at 60 °C for 50 minutes, using a water bath as the heat source. This “plug” also served as the elution section, where the DNA was released from the beads and detected using a hand-held UV-flashlight to reveal green fluorescence.^15^

**Fig. 2:**
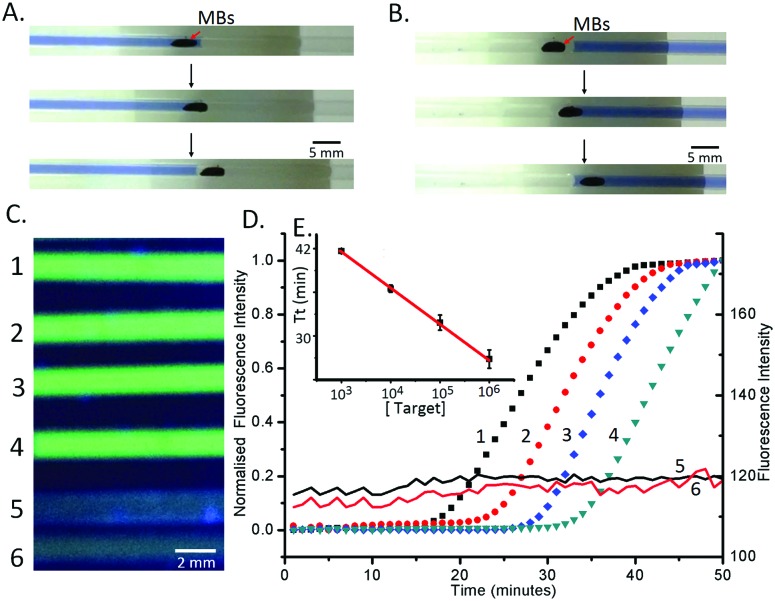
Continuous images showing, under the influence of magnetic field, the movement of MBs (red arrow) between solutions through immiscible mineral oil. (A) The movement of MBs from water (blue) to mineral oil (white); (B) the movement of MBs from oil (white) to water (blue). Pictures were taken using a smartphone camera and an LED ring lighting from above. (C) Examples of fluorescence images of the LAMP mix plug containing different concentrations of *E. coli* carrying the target DNA as a plasmid, at the end of capillaries illuminated with a UV flashlight (from the bottom) and captured using a smartphone camera. (1) 2.5 × 10^6^ bacteria per ml; (2) 2.5 × 10^5^ bacteria per ml; (3) 2.5 × 10^4^ bacteria per ml; (4) 2.5 × 10^3^ bacteria per ml; (5) 2.5 × 10^2^ bacteria per ml. (6) ddH_2_O negative control; (D) real-time amplification curves (1–4 normalised, left axis), and ddH_2_O as a negative control (5 and 6, right axis, not normalised to ease readability): (1) 2.5 × 10^6^ bacteria per ml (black square); (2) 2.5 × 10^5^ bacteria per ml (red circle); (3) 2.5 × 10^4^ bacteria per ml (blue lozenge); (4) 2.5 × 10^3^ bacteria per ml (green triangle); (5) 2.5 × 10^2^ bacteria per ml (black line); (6) negative control (red line). (E) Threshold time (defined as the time corresponding to 50% of the maximum fluorescence intensity, *T*_t_) as a function of target concentration. Data are the average of at least 3 replicates and error bars represent the standard deviation. The data were fitted with linear regression (*R* ^2^ = 0.999).

The results of the LAMP amplification were directly read out visually, and we established a limit of detection of the capillary-based assay by processing ten-fold serially diluted cultured *E. coli* (Promega), which contained the conserved fragment of *C. trachomatis* at 2.5 × 10^3^ bacteria per ml (Fig. 2C and D), corresponding to 2.5 copies of the plasmid in 1 μl. This approach was validated using a real-time (RT) amplification approach, where the signal was quantified using fluorescence microscopy^4^ (Fig. 2D). As the target concentration increased, the exponential phase of signal enhancement started earlier, from *ca.* 18 minutes for 2.5 × 10^6^ bacteria per ml to 33 minutes for 2.5 × 10^3^ bacteria per ml.

To assess the efficiency of the capillary system, we used the threshold time (*T*_t_) (Fig. 2E), as the reaction time for the fluorescence signal to reach 50% of the maximum,^4^ which shows a linear decrease with increasing target concentration (on a logarithmic scale). The value of *T*_t_ is important as it enables us to quantify the original amount of target DNA present in the reaction after calibration, opening up applications in optimising medical treatment such as those based on viral load quantification.^16^

Contrary to more complex DNA amplification techniques, such as PCR, LAMP does not require precise thermal control or cycling of the reaction (previously LAMP has been implemented using pocket warmers^6^ and thermos containers^17^). Here we simply poured hot water (*ca.* 300 ml at 64 °C) into a thermally-insulated polystyrene foam container providing the required stability over 1 h, with the temperature only cooling down to 60 °C over this period (Fig. 3A). The only external energy required for the LAMP was the warming of the water, which can be easily performed even in remote locations. The functionality of the approach was compared with a controlled incubation at 63 °C, using gel electrophoresis analysis of the amplified products (see the ESI† for Experimental details), and showed no appreciable difference (Fig. 3B).

**Fig. 3:**
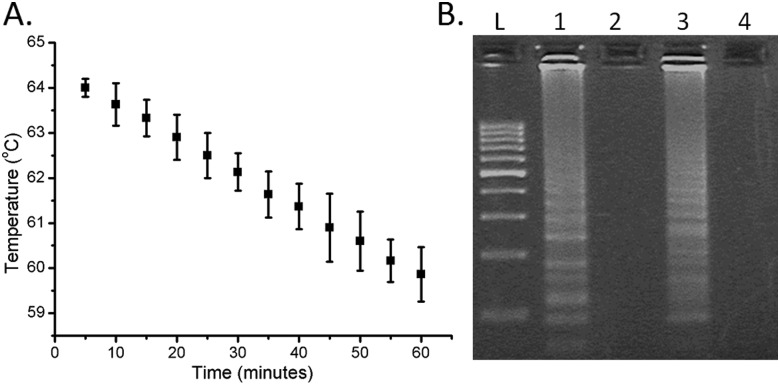
(A) Temperature variability within the insulated chamber. *ca.* 300 ml of water at 64 °C was poured into the chamber and monitored for 60 min. Data are the average of at least 3 replicates and error bars represent the standard deviation. (B) Agarose gel results of *N. gonorrhoeae* within the capillary platform. Lane L 100 bp ladder; lanes 1 and 3 were positive and lanes 2 and 4 were negative. Lanes 1/2 were the reactions kept at 63 °C constantly, while lanes 3/4 were incubated in the chamber. No appreciable difference between the two systems is visible.

To demonstrate the multiplexed capability of our capillary platform, an additional LAMP reaction mixture plug targeted at the detection of *N. gonorrhoeae* (see Methods in the ESI† for its composition) was added after the plug targeting *C. trachomatis*, into a single capillary, separated by mineral oil. Due to the sequential nature of the elution in the two plugs dividing the DNA contents, the volume of the second plug (*N. gonorrhoeae)* was reduced to 3 μl, compared to that of the first one (*C. trachomatis* – 5 μl) (Fig. 4A). After amplification, the fluorescence signal in the two sections of each capillary indicated the presence of the targeted sequence of each pathogen simultaneously (Fig. 4B).

**Fig. 4:**
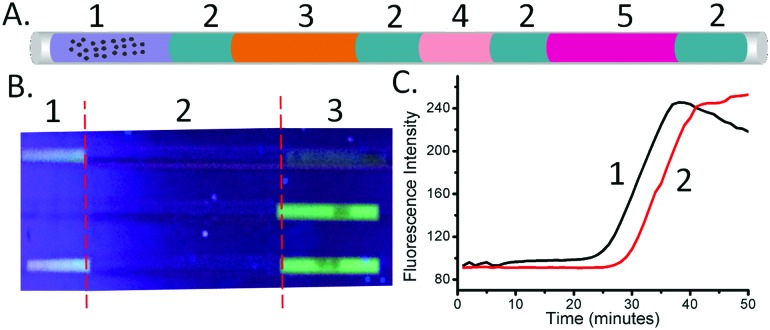
Multiplex LAMP assay. (A) Multiplex LAMP system with preloaded reagents. (1) Lysis buffer with MBs; (2) mineral oil; (3) washing buffer; (4) LAMP reaction reagent 1 (3 μl); (5) LAMP reaction reagent 2 (5 μl); (B) simultaneous detection of *C. trachomatis* and *N. gonorrhoea*: (1) *N. gonorrhoea* LAMP mix; (2) mineral oil; (3) *C. trachomatis* LAMP mix. Three capillaries were measured with different samples (from the top to bottom): *N. gonorrhoea*, *C. trachomatis*, and *C. trachomatis* and *N. gonorrhoea* sample simultaneously. The pictures were obtained using a smartphone camera and illumination with a UV flashlight (from the bottom). (C) Real-time LAMP amplification curves of the reactions with an initial concentration of 2.5 × 10^5^ bacteria per ml, when (1) the beads are moved into the first plug (4 in A) and left there and (2) when the beads traverse the first plug and are stored in the mineral oil.

Finally, we further demonstrated the applicability of the technique in 6 patient samples, obtained as swabs and extracted as part of routine clinical diagnostics at the NHS West of Scotland Specialist Virology Centre. The results (yes/no indication of the presence of the pathogens) were consistent with those obtained using the benchmark assay real-time PCR (Table S1 and ESI†).

Using the ability of the real-time LAMP reaction to quantify the initial DNA concentrations (the amount of DNA present after extraction, before amplification – see Fig. 2), we also explored the influence of the retention time on the quantity of eluted DNA. When the beads were left in the first plug (4 in Fig. 4A), without moving the beads to the second LAMP plug (through the oil plug into 5 in Fig. 4A), the value of *T*_t_ was 30.5 ± 1.0 minutes. When the beads were moved through the first plug into the mineral oil, the *T*_t_ value for the LAMP mix of the first plug was 35.5 ± 0.5 minute. Using the equation derived from Fig. 2C, we calculated that the recovery of DNA when the beads only pass through the plug of LAMP reaction mix (4 in Fig. 4A) was 15% ± 5% of that when the beads are left in the plug.

In conclusion, in this study, we have reported a capillary-based, magnetically-actuated, multiplexed LAMP assay platform for the rapid diagnosis of *C. trachomatis* and *N. gonorrhoea*. Its simplicity, low-cost and low energy requirement (only a tea cup volume of hot water required) holds significant promise for its application in low resource settings.

This work was supported by a College of Science and Engineering Studentship (GX, Glasgow, UK), a Lord Kelvin and Adam Smith Research Fellowship (JR, Glasgow, UK), an EPSRC fellowship (JC, EP/K027611/1), and an ERC Advanced Investigator Award (JC, 340117). All the original data related to this article are within the depository of the University of Glasgow with http://dx.doi.org/10.5525/gla.researchdata.349. Additional data related to this paper may be requested from the authors.
