# Supplementary material for: A capillary-based multiplexed isothermal nucleic acid-based test for sexually transmitted diseases in patients
Source: Chem Commun (Camb). 2016 Sep 8;52(82):12187–90. doi: 10.1039/c6cc05679b (PMC5058349; doi:10.1039/c6cc05679b)
Supplement: Supplementary file 1 [file CC-052-C6CC05679B-s001.pdf]

Electronic Supplementary Material (ESI) for ChemComm.
This journal is © The Royal Society of Chemistry 2016

Capillary-based multiplexed isothermal nucleic acid-based test for sexually transmitted diseases in patients

Gaolian Xu; Hang Zhao; Jonathan M. Cooper and Julien Reboud

Supporting information

Experimental Section

LAMP reaction. The LAMP primers and plasmids containing the conserved nucleic acid fragment of *Chlamydia trachomatis* and *Neisseria gonorrhoeae* respectively were constructed based on our previously study, [1] where the sequences are available. Briefly, the highly conserved fragment of *cppB* gene of *N. gonorrhoeae*[2] and cryptic plasmid of *C. trachomatis*[3] which is present at approximately 5 copies per bacterium were used for LAMP primer design, respectively. Four primers, comprising two outer (F3 / B3) and two inner (FIP / BIP) primers were designed using LAMP primer software Primer Explorer V4 (http://primerexplorer.jp/elamp4.0.0/index.html; Eiken Chemical Co., Japan). Primers were tested for self-dimer, hetero-dimer and self-hairpin structures using the Integrated DNA Technologies design tools (http://eu.idtdna.com/pages/scitools). The loop primers (LFP/LRP) were designed manually. The *E.coli* containing plasmids served as LAMP amplification targets. The primer concentrations for the LAMP reaction were 1 µM (FIP/BIP), 0.8 µM (LFR/LRP), and 0.1 µM (F3/B3). The LAMP reaction mixture also contained 0.4 mM of each dNTPs (Sigma), 4.0 mM of $MgSO_4$, 50 mM Tris-HCl (PH 8.1), 30 mM KCl, 30 mM $(NH_4)_2SO_4$, 0.1% Triton X-100, 1 M of Betaine, 25 µM Calcein (Sigma), 500 µM $MnCl_2$ and 8 units Gsp SSD DNA polymerase (OptiGene, UK). The whole reaction was incubated at 63 °C for 50 minutes.

Materials. The MagaZorb DNA Mini-Prep Kit was purchased from Promega to assemble the LAMP system with the magnetic rack from GE healthcare. More specifically, it contained the magnetic beads used in the capillary format. These were 4.5 µm in diameter, treated with 10% PEG (w/v),[4] and were used as recommended by the manufacturer at a concentration of 50 mg/ml. The 152 mm long glass capillaries (1B150-6) with an inner diameter of 0.84 mm, an outer diameter of 1.5 mm were purchased form World Precision Instruments, Inc. The S25234 silane (2-[Methoxy(polyethyleneoxy)propyl]-trichlorosilane) was from Fluorochem Ltd. (UK). Epoxy glue (5 minutes) used to seal the ends of capillaries with quick curing properties was from Devon. Guanidine thiocyanate (GUSCN) was purchased from Sigma. A hand-held UV light with wavelength at 365 nm was used for the amplification product readout.

Capillary preparation. For the LAMP system, the capillary was treated with 20µl of silane (S25234) as purchased for 30 minutes to obtain a hydrophilic inner surface in a similar fashion as Kim et al.[5] After treatment, the capillaries were rinsed three times with distilled water and dried with N_2 before use. The following plugs were manually inserted sequentially into the capillary using a 10 µl pipettor : lysis buffer (4 µl, 4 M GUSCN, 25 mM sodium citrate, 0.2% sodium dodecyl sulphate),[6] also containing the magnetic beads (MBs), washing buffer (10 µl), LAMP amplification reagents (10 µl) and mineral oil (10 µl), as shown in Figure 1A of the main text.

Fluorescence Measurement. The real-time fluorescence intensity of LAMP reactions was also quantified using a fluorescence microscope (Axio Scope A1, Zeiss) with a 5x objective and a FITC filter set (490 nm excitation, 515 nm emission). The reactions were observed directly *in situ* in the capillaries without any manipulation. The data was quantified using the Wasabi software (Hamamatsu Photonics) and was collected every minute.

Gel Electrophoresis. The products of the reaction were analysed in 2% agarose gels with SYBR gold (Invitrogen) in 1× Tris/Borate/EDTA (TBE) buffer (Invitrogen). Gels were run at 70 V for 120 min and visualized under with SYGENE PXi.

Clinical sample validation. Six clinical suspected infected patients were used to test the validation of our assay. These were swab samples from potentially infected areas gracefully provided by West of Scotland Royal Infirmary Clinical Diagnostics Laboratory (WoSRICD). They were extracted following the clinical standard operating procedure of the WoSRICD by using the QIAamp DNA Mini Kit from Qiagen and analysed using a commercial assay (Abbott RealTime CT/NG assay, Abbott Laboratories, USA), following the standard operating procedure in place. Of the six samples, one was *C. trachomatis* positive, two were *N.gonorrhea* positive, two were all negative. One sample (sample 4) tested positive *for N. gonorrhoeae* and *C. trachomatis* simultaneously (Table S1 below).

Table S1. Clinical samples characterization, benchmarked against the standard real-time PCR assay (showing Ct when available)

Sample #	*C. trachomatis*		N. gonorrhea	
	Real-time PCR	Multiplexed capillary LAMP	Real-time PCR	Multiplexed capillary LAMP
1	negative	negative	24	positive
2	negative	negative	29.48	positive
3	25.68	positive	negative	negative
4	30.68	positive	27.48	positive
5	negative	negative	negative	negative
6	negative	negative	negative	negative

Additional References.

1 G. Xu, R. N. Gunson, J. M. Cooper and J. Reboud, Chem. Commun., 2015, 51, 2589–2592.
2 D. J. Farrell, J. Clin. Microbiol., 1999, 37, 386–390.
3 R. Marshall, M. Chernesky, D. Jang, E. W. Hook, C. P. Cartwright, B. Howell-Adams, S. Ho, J. Welk, J. Lai-Zhang, J. Brashear, B. Diedrich, K. Otis, E. Webb, J. Robinson and H. Yu, J. Clin. Microbiol., 2007, 45, 747–751.
4 US Patent 6855499, 2005.
5 S.-H. Kim, J. W. Kim, J.-C. Cho and D. A. Weitz, Lab. Chip, 2011, 11, 3162–3166.
6 A. Creecy, P. K. Russ, F. Solinas, D. W. Wright and F. R. Haselton, *PloS One*, 2015, **10**, e0130260.
